# The Role of Food Literacy in Managing Nutritional Precarity in the Migrant Experience: Dietary Lifestyles of Cambodia Migrants in Thailand

**DOI:** 10.3390/nu14245380

**Published:** 2022-12-18

**Authors:** Sary Seng, Hart Nadav Feuer, Sayamol Charoenratana

**Affiliations:** 1 Human Security and Equity Research Unit (HuSE), Social Science Research Institute (CUSRI), Chulalongkorn University, Bangkok 10330, Thailand; 2 Graduate School of Agriculture, Kyoto University, Kyoto 606-8501, Japan

**Keywords:** food literacy, precarity, COVID-19, migration, Thailand, Cambodia

## Abstract

The paper explores the dietary lifestyles of young Cambodian migrants in Thailand to illuminate the role of food literacy in determining nutritional outcomes and well-being, including during crises, such as the COVID-19 pandemic. In this context, food literacy is defined as food skills and abilities to plan, select, and prepare to achieve adequate consumption under new or adverse social and culinary contexts of the migrant experience. In this paper, we consider both how nutritional precarity arises in the migrant experience, and to what extent food literacy can mitigate it under various conditions. The research approach involves a combination of qualitative and quantitative approaches that were adjusted to address the limited mobility for social science research during the COVID-19 pandemic in Thailand. Data collection was conducted through hybrid (online and in-person) ethnography, focus group discussions, food literacy questionnaires, and key informant interviews, often facilitated through internet messaging clients. The findings indicate that, while generally high food literacy may facilitate the transition to the foreign food systems found in migration destinations, optimizing nutrition and well-being requires reinforcement by context-specific food literacy, such as openness to foreign flavors and recipes. Contextual food literacy most directly leads to positive social and health outcomes while simultaneously expanding universal food literacy in the long-term.

## 1. Introduction

The goal of this research is to determine the extent to which food literacy has facilitated survival or improved dietary outcomes among migrants facing precarity, including those encountering unique challenges under the COVID-19 pandemic. The paper contributes to the academic literature on human security and equity, with a focus on managing challenging nutritional conditions under the varied conditions of migration. Food literacy has increasingly been raised as an important dimension in resolving food systems precarity of many forms in recent decades [1,2,3,4]. Indeed, the landmark study for defining food literacy centered on the experiences of young people managing their health and well-being while living in disadvantageous conditions [5,6]. In general, food literacy has become a popular term to explain the interplay between individuals (agency) and their food environment (structure). While the basic understanding of food literacy’s evolving contribution to health and well-being focuses on usual lifecycle variations, including childhood, emancipation, career, and homemaking [1,7,8], it is more poorly conceptualized for individuals who cross national/culinary borders or live in extreme conditions (such as on deep-sea boats, in work camps, or isolated places). Indeed, one of the most significant challenges facing efforts to develop global standards for food literacy measurement has been differences in national food systems and cultures, or more generally, the need for significant localization and adaptation [5,8,9,10,11]. This sticking point in the literature raises a fundamental question: To what extent can food literacy be considered a universal skill, rather than a contextually embedded skill?

The premise of this paper is the assumption that some or even many aspects of food literacy have broad utility and can be adapted to radically new contexts, while other aspects might even complicate or impede adaptation to new contexts. For example, basic food preparation skills (such as chopping, frying, steaming) might readily facilitate adaptation to a new food environment, others (such as baking) can become redundant or distracting in contexts lacking specific equipment, and yet others (such as tendency to buy regularly from fresh markets) can complicate life in a supermarket-based society. In this paper, we take up the case of Cambodian migrants to Thailand, who are faced with both a moderately different culinary system and radically different food environment centered around construction camps, factory slums, and deep-sea fishing boats. We evaluate the extent to which food literacy can create a basis for dietary resilience in the precarious food environments facing international migrants.

**Figure 1:**
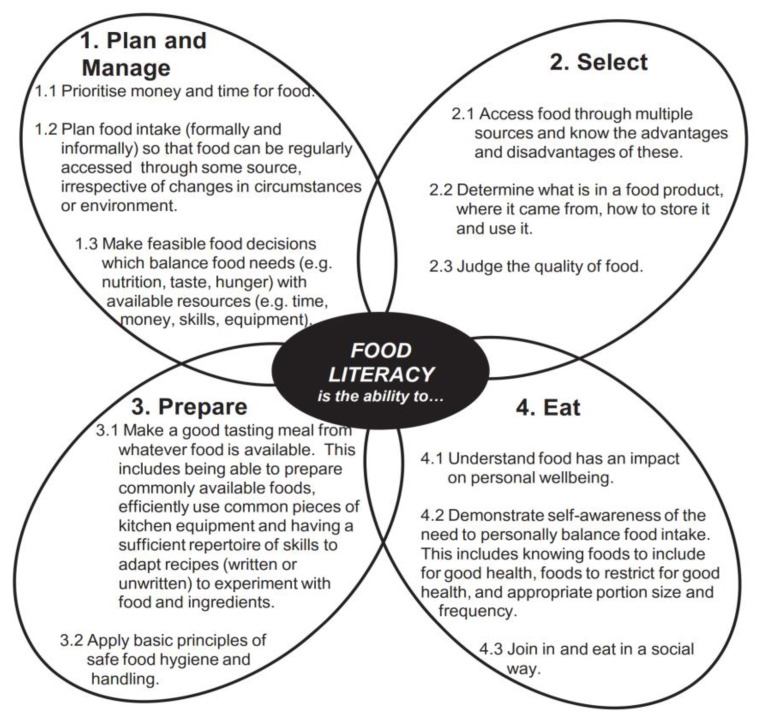
Food Literacy pillars and components (Source: Vidgen and Gallegos, 2014).

As laid out by Vidgen and Gallegos [5] in their reference publication on the components of food literacy, a person’s ability to find food, plan for consumption effectively, and prepare a nutritious diet for themselves, are lifelong skills that are revised to suit the varied food environments that people face throughout their lives (see Figure 1). In fact, Vidgen suggests that food literacy “has been conceptualized as supporting resilience” [6]. In principle, the pillars of food literacy shown in Figure 1 are understood to be “meta skills” that allow individuals with high food literacy to readily engage with an unfamiliar food system around them. For example, someone who is adept at eating whole fish in Norway would be expected to quickly master the consumption of different varieties of fish found in the Philippines, and vice versa. However, it is less clear if a good meal planner from Norway, who has never lived in a tropical country without a refrigerator, rigorous hygiene regulations, and supermarkets, would manage in a Filipino village with only fresh markets and limited refrigeration. Similarly, a skilled Filipino meal planner accustomed to shopping daily at fresh markets, might struggle to envision stocking a week’s worth of groceries from the supermarket in a refrigerator. In fact, we argue that the individual components of each food literacy pillar (those within each petal in Figure 1), such as familiarity with local ingredients, recipes, equipment, means of cooking, and language-specific labels, often rely on contextual, embedded knowledge. Considering this, the more one faces divergences from a familiar food environment, the more challenging will be the adaptation regardless of one’s level of food literacy. With this in mind, we turn to the case study of this paper: Cambodian migrant workers engaging with the food environments they encounter in Thailand.

Many studies have concerned themselves with the dietary well-being and nutritional status of migrants in host countries, which can include long-term migrants as well as short-term ones, such as students and laborers [12,13,14,15,16,17]. These studies have largely focused on the outcomes of displacement on health and culinary composition, and often reference the major disparities between the food systems of the host and sending countries as an explanatory factor for sub-optimal adaptations (e.g., eating junk food) or poor outcomes (e.g., obesity) [13]. Although publications on this topic have occasionally referenced the food skills or domestic skills of migrants, in general the pathway through which dietary health is impacted by migration is undertheorized. As Vidgen [6] proposes that food literacy can improve nutritional resilience, studies about the role of food literacy in dietary adaptation in host countries would be a welcome contribution to resolving this research dilemma. This study sits squarely in this domain, exploring the question of how food literacy can be engaged to compensate for precarious living conditions, including limited access to markets, unfamiliar ingredients, and inadequate cooking equipment.

Under such precarity, having skills that help to survive or manage unstable living and working conditions can be considered an asset that facilitates local integration or improves well-being [18]. Migrants, whether urban–rural or international migrants, often face dietary precarity at some stage of their journey, and thus departing with high food literacy might be understood as good preparation. Having adequate agency to compensate for unstable or adverse structure in the food system has been theorized to help manage moments of nutritional precarity in migration [19]. To address this, we ask how a food literacy approach, which principally focuses on individual agency, can accommodate or take account of structural factors, such as new food environments, living conditions, and socio-economic status?

To understand how food literacy is used by migrants to adapt to variable conditions, it is important to understand the degree to which pre-existing food literacy is relevant in the new environment. Relevance depends on the correspondence between the new food environment and previous ones concerning food access, composition (ingredients), equipment, and social environment [2,6,7,20]. The question we ask is to what extent seemingly unrelated or divergent food environments can engender transferable or adaptable food skills, and what factors might explain the expansive utility of food literacy. Young people who have grown up in Cambodia will, depending on their upbringing, have varied experiences from which to draw on when they move to Thailand for work, some of which will be directly applicable to their new environment, while other cases may require improvisation. In this model, rural children who grew up scavenging, catching fish, and preparing food without “proper” cooking equipment may be able to leverage these skills as adults in adverse food environments, such as migrant living spaces. In short, a range of important food literacy knowledge is often indirectly or informally learned, which may emerge as a useful asset in the future [2].

International migrants who are disconnected from kinship resources, in a foreign food environment, or facing difficult working conditions, are put under extreme pressure to feed themselves in a balanced manner—much in the same way that poverty or slum life affects people’s abilities to achieve reasonable nutrition [3]. For Cambodians, Thailand is a common host country for migrant workers; it also shares a similar food system and cuisine. Approximately 5 million migrant workers are in Thailand, of which 2.3 million are formally registered [21]. Among the 1.8 million Cambodian migrant workers, however, only 231,151 are formally registered, which indicates that most Cambodians are in vulnerable and precarious work conditions [12,21]. Although cumulative migration networks and familiarity with the destination have eased conditions, or at least created more realistic expectations, the living conditions remain harsh in places such as construction camps or on deep sea fishing boats [22,23]. During the COVID-19 pandemic from 2020, Cambodians faced new challenges to their mobility, access to markets, and opportunity for sharing resources through social eating. A drop in remittances and starkly reduced migratory flows occurred in parallel to the closure of food services due to hygiene measures, making the food system in Thailand (even) less affordable and accessible [21]. By covering not only the challenges faced by migration and precarity, but also those prompted by the COVID-19 pandemic (unemployment, social exclusion, and vagrancy), many dimensions of past studies of youth poverty that inspired the current food literacy model are also addressed [5,6].

## 2. Materials and Methods

The following research approach was adopted to study spaces of migrant workers in Thailand, in which Cambodian migrant workers are found. Many of the methods listed here were designed to accommodate the evolving restrictions for in-person research during the COVID-19 pandemic. Fieldwork was conducted by a Cambodian national who speaks Thai and Khmer, and who has extensive ethnographic experience working with vulnerable populations. Initial contact was made through migrant networks, and thereafter informants were snowball sampled with a goal of representing a wide range of the migrant experience in Thailand, and across the three sectors (factory, fishing, construction). Ethics approvals for this research were conducted within a specialized university research institute focusing on human equity and human security.

### 2.1. Online and In-Person Ethnography

Through direct observation or video chats of migrant residences and worksites, the living conditions, food environment, and personal/communal dietary practices were observed. The purpose of this method was to grasp the range of dietary lifestyles of migrant workers and consider these in light of different forms of precarity (illegal status, poor housing, COVID-19 mobility restrictions, etc.). The range of dietary lifestyles found in the three different sectors were analyzed in an exploratory way using inductive coding and subsequently categorized in food literacy terms. Through this, we determined how, and in which ways, migrants are coping with various levels of precarity in their job sites and/or slums. A subsidiary goal of this phase of exploratory research was to gather information to help refine a quantitative survey of food literacy, which would map how life skills facilitating survival during precarious times are related to the different pillars of food literacy (planning, selection, preparation, and consumption). Understanding the range of skills through observation, and exploring the origins of these skills through interviews, helped to develop a more reliable assessment of the utility of different aspects of food literacy. After this stage, the role of food literacy was explored through selected focus group discussions (FGD).

### 2.2. (Online) Focus Group Discussion

The FGDs focused on producing a broader narrative about the use of food literacy (including both overt and soft skills) in facilitating survival or crisis management during the COVID-19 pandemic. These FGDs sampled Cambodian migrant workers from destinations where precarity is generally high, but was exacerbated by the closure of the Thai-Cambodia border due to COVID-19. Three sector-targeted FGDs were conducted in Khmer language, representing 5 participants from the construction sector, 5 from fishery work, and 5 from factory work. All informants were selected based on recommendations provided through the informant network developed in previous research stages. Given that this was a closed network of trusted individuals, it is possible that some participants may know one another or be from the same place of origin. Participants’ demographic characteristics, including gender, age, and educational backgrounds, were measured to make considerations for socio-economic background. To control for food literacy development throughout life, only young migrant workers under the age of 18 were considered for FGDs. Because of pandemic traveling and meeting restrictions, Zoom or group video chat were used to conduct the FGDs. The recordings were coded directly using a deductive approach (i.e., the food literacy scheme) to illuminate consensus and divergence about the utility of past food literacy in each respective context.

### 2.3. (Remote) Survey of Food Literacy

After the first two stages of qualitative research, a questionnaire to benchmark individual food literacy was designed and implemented. The questionnaires create a baseline of food literacy among respondents, from which evaluation of its utility can be reflected on, in light of the qualitative data collected in other research stages. The main purpose of the (online or remote) survey was to measure how the four pillars of food literacy were adapted to the life and needs of migrant workers, many of whom were stranded due to COVID-19 or in other precarious positions. The food literacy measurement we used was outlined in Perry et al. [11] and amended by Amouzandeh et al. [24]. To achieve a high level of reliability and credibility in the results, the questionnaire and interview guide were tested for accuracy, word choice, and comprehensiveness before being employed. The survey was conducted with 100 young Cambodian migrant workers. These participants were drawn in equal proportion from construction work in Bangkok, fishery work in Rayong Province, and factory work in the outskirts of Bangkok. The questionnaires were made available in Khmer. To guarantee respondent safety, no personally identifiable information (PII) was requested, and data were analyzed in an aggregated form. The survey results are presented in a descriptive way only, with context and meaning substantiated through qualitative results.

### 2.4. Elite Informant Interviews

An in-depth interview approach was used to gather insights from stakeholders who are knowledgeable about, or close to, migrant workers. Five key informants representing civil society organizations, employers, and migration brokers, were interviewed by telephone or video chat. Informants were chosen who could speak to the relative capacities of different workers to manage under precarious situations—and to the strategies employed by those with different backgrounds and (food literacy) skills. These interviews were analyzed primarily with the intention of triangulating observations and insights from migrants themselves.

## 3. Results

The results of this study are presented thematically, with primary data synthesized and integrated into key conceptual blocks that correspond to the central research question concerning the utility of food literacy in the migration experience. The initial findings begin with pre-migration food literacy, proceed to contextual adaptation in different sectors or geographic spaces, and finally present key domains of food literacy that play a role in well-being or health of Cambodian migrants in Thailand.

### 3.1. Advantages of Diverse Sources of Food Literacy

Food literacy is at once contextual—corresponding to the norms of local cuisine, agriculture, and markets—and, on the other hand, universal in that it always requires adaptation and improvisation to the vagaries of seasonality, market fluctuations, and budget, as well as individual preferences. While a migratory experience within the same region or between similar culinary systems, such as Thailand and Cambodia, may not render contextual food literacy ineffective, more universal skills can more readily be engaged upon encountering unusual lifestyle patterns and food systems faced abroad. Cambodian and Thai cuisine, for example, share a similar basis in sub-tropical rice agriculture that focuses on freshwater fish, soups, aromatic herbs, and fruit [25,26,27], while systems of distribution for fresh produce and meat, including wet markets, are also similar. This correspondence facilitates some transfer of contextual food literacy, such as recipes, cooking styles, and familiarity with ingredients. However, migration destinations such as work camps, coastal fishing ports, and slums often constrain or warp the way in which the broader food system can be encountered and experienced. For example, the availability of, and access to, markets can be impacted by geographic factors and/or restrictions on mobility faced by migrants, while intensive work lifestyles can hinder familiar patterns of grocery shopping, preparation, and consumption. Differences in infrastructure can also be decisive, such as available cooking equipment, food storage possibilities, and access to dedicated food preparation spaces (for smokey, grilling, or butchering). Migrants with more universal food literacy will have an easier time adapting to divergences in conditions for food acquisition and food preparation.

Broadly, migrants with more diverse sources of food literacy prior to migration are more likely to have developed both universal and contextual capacity for adaptation to migration destinations. In this sense, an assessment of young Cambodian migrant worker’s food literacy must explore the background and knowledge they have gained in their home country. Most migrant workers (including in our sample) have a rural background in Cambodia and are thus familiar with overall trends of seasonal food acquisition, fermentation, low-tech food storage, and gaps in food availability that may require rationing. They often have some skills in gathering wild food, including herbs, fish, fruit, and alternative protein sources (insects and other non-mammals). Most of the informants in the survey (75% or 80% in different dimensions) acknowledged that they were confident to convert any seasonable vegetable or protein into a meal, with women expressing somewhat more confidence than men. In FGDs, respondents widely agreed that their rural upbringing gave them personal dietary resilience: they could endure periods of food insecurity or eat monotonous food for long periods without complaint (the latter is particularly useful for fishermen on long trips). Overall, rural migrants may have extensive abilities to discern freshness (including in local Thai markets) and high appreciation of homestead agriculture (non-commercial varieties of vegetables and proteins), but less familiarity with urban market skills, such as the evaluation of pesticide residues and hygiene.

“I grew up in a village in Pursat province so I knew all about the farm seasons, which vegetables are fresh and how to cook different fish and other things like snails. The family spent a lot of time preparing food and I got a lot of practice. But honestly I had a difficult time when I came to Thailand at first. It is so different shopping here and finding fresh food at a good price. You need to learn a lot about the market and you need to have more time to shop and cook. I think I don’t always have time to do a good job cooking.”

(female, age 23, factory slum, 6 September 2021)

Rural migrants also reveal limited awareness and skill in evaluating processed food products, often being unfamiliar with significant points such as expiration dates, synthetic ingredients, or compromised packaging (such as broken cans). In general, migrants with life experiences outside of their hometown or family life—particularly urban living—have often developed a wider set of skills that help to navigate the challenges of migration destinations [3].

Young people with no prior migration or even urban living experience in Cambodia are often the most disadvantaged when transitioning to Thailand. They are confronted simultaneously with the general challenges of managing in urban food systems as well as the unique lifestyle and food environments of migration destinations. An advanced understanding of the economic and agricultural uniqueness of urban food markets prior to migration instills a broader set of skills around planning, economical shopping, and food safety. Familiarity with the urban landscape of restaurants, food stalls/vendors, as well as supermarkets and the role of processed food and ingredients, provides critical insight about how to balance nutrition and convenience in an economical way.

“I was a factory worker in Phnom Penh before I came to Thailand. I learned a lot from my time there; my cousins taught me so much about how to get food fast and cheap from the daily market, how to choose the right food to cook in our small room, and how to prepare lunch for the next day. You have to think about this every day to do a good job or else you end up spending money at food stalls or eating junk food.”

(female, age 22, factory slum, 30 August 2022)

**Figure 2:**
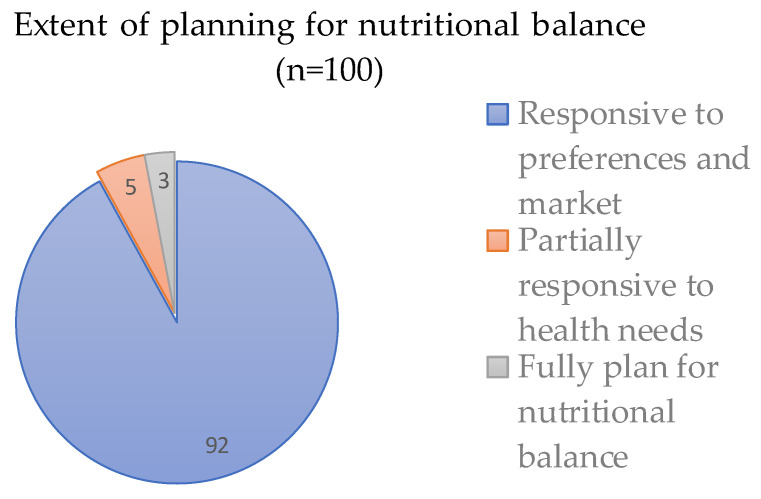
How survey respondents report balancing market needs against health and nutritional needs when grocery shopping.

Prior experience cooking in urban settings such as indoor kitchens or even cramped slums provides practice in food preparation and unique insights into urban meal planning and dining. A key strategic orientation of dietary planning in migration destinations is balancing long-term health impacts against everyday economical and personal considerations. Figure 2 reveals that most migrants prioritize daily preferences/cravings and cost over long-term nutritional needs.

“I do the best I can buying food to stay healthy without spending too much money. But if I’m honest, mostly I go day-by-day to eat what I want or what others in the house want. Maybe if I feel sick, or my skin starts to look bad, I’ll try to eat better for a while.”

(male, age 19, construction camp, 10 September 2022)

Part of the narrative in the quote above relates to the temporality of migration. Few workers explicitly desire or plan to have a lasting sojourn abroad, so long-term health considerations are not often entertained until health problems emerge. Consequently, among survey respondents whose dietary planning prioritizes health and nutrition (see Figure 2, smaller pie slices), they often had prior experience in urban living, or the period of sojourn abroad tended to be longer.

In summary, while food literacy arising primarily from rural upbringing provides many indirect advantages related to food selection and preparation, urban living or urban work experience provide more direct skills and knowledge for thriving in many of the migration destinations.

### 3.2. Wider Food Literacy Facilitates Adaptation to New Culinary Context, Lifestyle, and Infrastructure

The different migration destinations in Thailand provide unique challenges to maintaining dietary health. Not only are worksites embedded in a (somewhat) foreign food system in Thailand, but they also feature unique sub-cultures related to the services, infrastructure, and work-focused lifestyles. The substantive differences between the different economic sectors are highlighted in Table 1. In many ways, inner-city construction sites with attached housing and factory areas with neighboring slums share many structural similarities associated with urban food systems, while suburban construction sites (often housing estates) feature some food system characteristics more familiar to rural migrants from Cambodia. During the COVID-19 pandemic, when many dense urban areas faced extreme restrictions on mobility, suburban work sites provided migrants with a few alternative means of food acquisition. Meanwhile, the food system associated with seaports and fishing boats, which are more sector-specific and sequestered, demanded rather distinct adaptations by migrants but also proved to be more resilient to quarantines and other restrictions imposed during the pandemic. The divergent features of these migrant destinations illuminate why and how various forms of food literacy can be engaged by Cambodian migrants in Thailand to improve their welfare and adapt to the idiosyncratic food systems in each context.

A first illuminating case study is that of the construction center in Bangkok, which reveals how context (rather than sector) manifest unique food literacy challenges. Construction sites in the metropolitan area are divided into two types depending on their relative location: (1) in the city center, centered around erection of high-rise buildings, and (2) on the outskirts of the city focused mostly on suburban housing estates or private homes. Typically, construction workers rely on four major food sources: wet markets, mobile vendors, wild foraging, and supermarkets. FGDs also revealed that, for workers in suburban sites, it was also possible to gather food in the adjacent environment, including vegetables, fruit, and non-mammal protein (fish, shellfish, insects, amphibians, etc.). During the COVID-19 pandemic, construction was largely suspended, with workers laid off and mobility out of housing camps restricted by lockdowns. Only contractors, medical officers, and humanitarian workers were able to enter to distribute foods and conduct medical procedures. Food donated by the Thai government, contractors, and humanitarian NGOs became one intermittent source of food. Otherwise, the food systems challenges arising from lockdowns and loss of income diverged depending on the location. Restrictions on mobility effectively curtailed conventional food sources for construction workers, such as markets and mobile food vendors, for some periods, leaving only wild gathering or bartering as options for food acquisition. Donations met basic needs for various periods but the importance of self-provision, often through wild gathering, became very common. Migrants were able to directly engage food literacy derived from rural upbringing in Cambodia and, even after lockdowns concluded, gather wild food to increase their enjoyment and meet nutritional needs.

“When I was young, I used to collect food around the village and school for fun, or to make my parents happy. I collected fish, frogs, snails, fruit high up in trees, medicinal herbs, and when I was older I could catch snakes and insects. I never realized that I would end up doing the same thing in Thailand to help feed my family, but now some food I collect is the only thing we have some days.”

(male, age 26, construction camp, 12 June 2022)

For migrants who live together as a family, which is common in construction camps, the female partner is typically obligated to manage domestic issues but, as focus groups revealed, during pandemic lockdowns, men often (re-)engaged skills in gathering wild food to compensate for limited food markets. A construction camp foreman confirmed that wild gathering was more common, sometimes disturbing nearby residents, but he hesitated to crack down during periods of food scarcity. The foreman recognized that it was “lucky” that his suburban worksite was adjacent to natural areas with wild food, including canals where fish live.

Factory workers and downtown construction camps, meanwhile, are more dependent on food retail, such as wet markets, mobile food vendors, and supermarkets. As most construction workers are undocumented (i.e., lacking a legal work permit), their hesitance to travel often means that they rely exclusively on the closest markets or local vendors. Although most factory workers have official work permits allowing mobility in Thailand, the long-term nature of factories and high concentration of workers means that local food markets are so well developed that there is little need for workers to venture far to acquire food. For different reasons, therefore, workers in urban construction sites and factory areas depend almost exclusively on nearby food retail. Understanding how best to make use of nearby food options, balanced against one’s work schedule, budget, and culinary skills, has engendered contextualized food literacy for navigating worksite food systems (as well as managing disruptions such as lockdowns). FGDs established that adaptations to workers’ dependence on nearby retail includes gaining resilience against the convenience offered by vendors, cultivating personal relationships with food sellers, and prioritizing lower-cost raw ingredients over processed ones.

**Figure 3:**
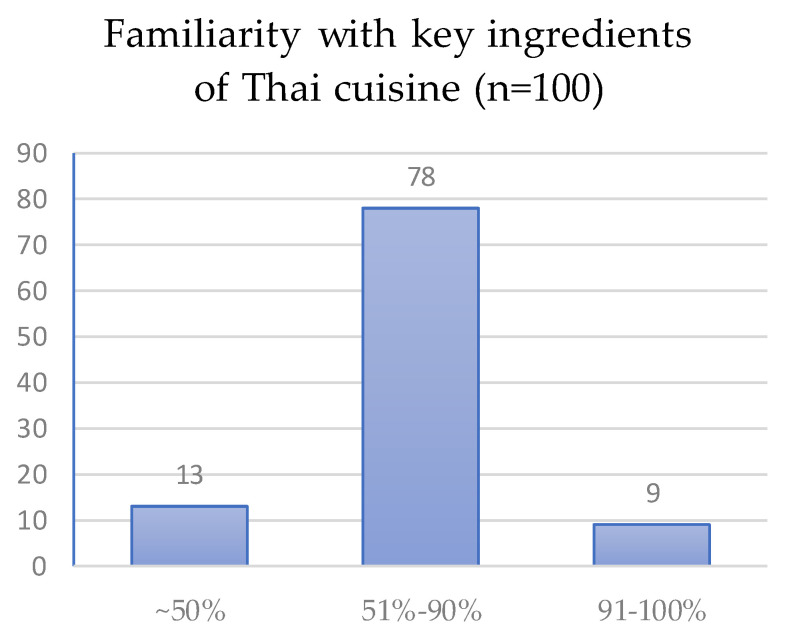
The percentage of key ingredients in Thai cuisine that respondents are able to use in their own cooking. In total, 78 out of 100 respondents report being able to competently cook with more than half (51–90%) of everyday Thai ingredients, which facilitates economical food planning.

One key long-term adaptation to reduce food costs is openness to Thai ingredients and recipes, which are more readily available and cheaper. As 70% of the survey sample reported that the main factors in their food selection were ability to cook the ingredient and price, expanding one’s culinary repertoire to Thai cuisine is a common approach among Cambodian migrants to save money (see Figure 3). This increases the range of possible ingredients, thereby increasing flexibility in meal planning and food purchase. Openness to new recipes was particularly salient among construction and factory workers in the sample, while fishermen often seek out ingredients for instrumental reasons, namely, to bring interesting ingredients onto the boats with them that are rarer on the open water, such as spices, livestock products, or durable vegetables (such as pickles), which help balance their diet. Upon returning to port, fisherman usually crave fresh vegetables, herbs, fruit, and wild (botanical) plants.

“I wasn’t a big vegetable eater as a child, but if I am on the boat for two weeks, I will be desperate to eat vegetables and fruit when I get back to the port. By now, I learned to love vegetables because I know how bad my body feels if I don’t eat them. But I’m also used to eating the same food every day on the boat—everyone gets used to it. I guess sailors all over the world face the problem of bad food.”

(male, age 18, port area, 10 June 2021)

In FGDs, fishermen recognize their strategic preference for fresh fruit and vegetables foods primarily from a hedonistic perspective (i.e., avoiding the monotony of fish and seafood on the open water), but there is also a nutritional logic that many recognize. Nevertheless, the skills to tolerate a monotonous diet and ration food, which were common during lean seasons in rural areas in Cambodia, have become helpful skills as fishermen.

### 3.3. Key Characteristics of Food Literacy for Cambodia Migrants in Thailand

One normative principle in the food literacy discourse is the potential to cultivate “soft” skills that help with adaptation to other food systems or contexts such as food poverty [7,9,21]. Critics of this optimistic view of food literacy view have argued that it places undue burden on individuals, when in fact much food-related adversity is structural in nature [28,29]. In this study, we found evidence for both views: highly food literate people may be better positioned to manage in a new or adverse food environment but contextual knowledge or embedded skills (e.g., about Thai cuisine, urban food acquisition, and work camp foodways) can offer decisive benefits as well. Based on an analysis of all data sources in this study (ethnography, FGDs, and survey), we mapped some key universal food literacy characteristics and some contextual ones that have proven salient among Cambodian migrants to Thailand. These characteristics can be classified under the four pillars of food literacy outlined in Vidgen and Gallegos [5] (see Table 2). Details about many of the characteristics mentioned in Table 2 can be found in the preceding sections, where they were elaborated.

One overarching finding from Table 2 is that, although contextual characteristics of food literacy can be numerous, many can be derived from more universal characteristics. For example, the universal characteristic under the Eat pillar “Interest to try new flavors” is obviously correlated to the contextual characteristic “Enjoyment of local flavors”. However, while interest in something such as “spiciness” may provide openness to new flavors, the endpoint “enjoyment of spiciness” (of Thai cuisine) is not a given—some Cambodian respondents still struggle to consume spicy food despite their best intentions. Over the long-term, however, the contextual characteristics developed during a (migratory) transition phase may become subsumed by the universal characteristic. To continue the prior example: learning to enjoy spicy food in Thailand will become a helpful universal characteristic if a migrant later works in India or Indonesia, where spicy food is also commonplace.

**Figure 4:**
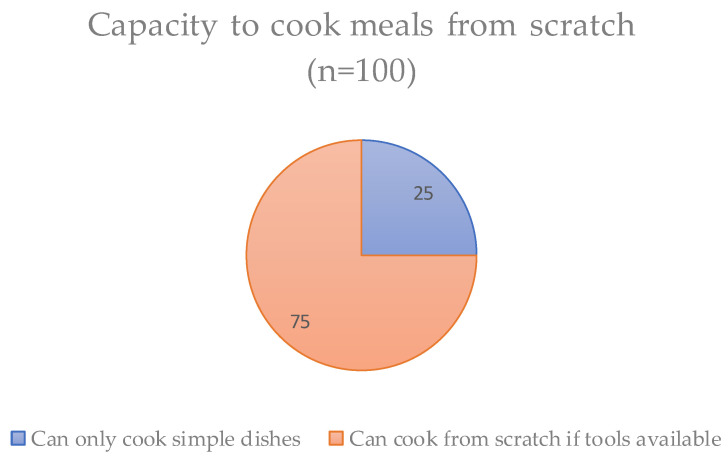
Degree of confidence in ability to cook a wide variety of meals from scratch.

Indeed, it might be concluded here that all universal characteristics of food literacy were originally developed as contextual characteristics. Within our survey sample, 25% of respondents claim that their cooking repertoire (even in Cambodia) is limited—they can only cook simple fried and grilled dishes and basic soups (see Figure 4). However, most of this sub-group is young, and therefore likely to learn how to prepare progressively more recipes until they no longer perceive themselves to have limited cooking abilities. Similarly, many Cambodian respondents who did not learn to appreciate wild foods while in Cambodia (including wild mushrooms, insects, snakes, turtle, frog, lizards, wild vegetables, etc.) have, through the dietary pressures induced by the pandemic, now highly value these foods. In short, food literacy is continuously expanding through new experiences or by overcoming adversity.

## 4. Discussion

Among the many challenges faced by labor migrants worldwide, adjusting to the new cuisine and food system in destination countries has been recognized but not comprehensively researched. Studies have documented the poor nutritional health outcomes of migrants in host societies [12,13,14,15,16,17], but there are few studies examining the evolution and reason for these outcomes. This study engages the perspective of food literacy to evaluate the relative agency and adaptability of migrants, centered around the case of Cambodian labor migrants to Thailand in the construction, factory, and fishery sectors. The discourse of food literacy has long been preoccupied with the capacity of people to achieve good dietary and nutritional outcomes when faced with adverse conditions, such as poverty or unhealthy food environments [1,20], and many proposed measurements evaluate adaptability and resilience [8,9,11,30]. This paper evaluates to what extent (pre-existing) food literacy, derived from experience in Cambodia, can be leveraged to avoid nutritional precarity when moving to another national food system and adverse context (work camps, slums, and fishing boats in Thailand). We ask how universal food literacy is by evaluating where it is engaged by migrants and where new, contextualized forms of food literacy must be cultivated in order to thrive.

Our study considers the background of Cambodian migrants and their long-term integration in Thailand, as well as their response to the severe restrictions imposed by COVID-19 pandemic lockdowns. We gathered primary data through hybrid (online and on-site) ethnographic means, focus group discussions, a food literacy questionnaire, and key informant interviews. The narrative that emerges from the long-term and proximate (pandemic) experiences is that food literacy has universal applicability in facilitating the transition to new food systems, but that developing more contextual food literacy associated with the local cuisine, markets, or lifestyle is a critical complementary process. Contextual food literacy, such as appreciation of local flavors, expansion of cooking repertoire, and familiarity with local ingredients (see Table 2) help to root—or embed—food literacy in new contexts. Ultimately, we conclude that contextual food literacy has both proximate utility in transition (such as migration experiences) and long-term potential for expanding and broadening one’s universal food literacy.

## Figures and Tables

**Table 1:** Sectoral and spatial differences in migrant destination food systems.

Sector	Idiosyncratic Characteristic	Shared Migration Characteristics
Urban (Construction and Factory)	Mobility restrictions for food acquisition (unrelated to the pandemic) Extremely cramped quarters and constrained cooking spaces Reliance on supermarkets due to rigid work schedule	Presence of vendors with migrant customers’ food preferences in mind (ingredients and processed food) Social eating and shared cooking enhance efficiency and create flexibility within groups Food preparation timing is limited by intensive work schedules Camaraderie with other Khmer migrants for ingredient acquisition Thai cuisine shares structural similarities with Khmer cuisine Thai wet markets and available produce are similar to Cambodian markets, except differences in pesticide residue Basic cooking equipment in Thailand matches Cambodia and is inexpensive
Suburban (Construction)	Access to open areas and wildlife (canals, undeveloped land) Opportunity for outdoor cooking and storage (BBQ, roasting, fermenting) Fair access to wet markets	
Seaport and Onboard (Fishery)	Discipline for eating monotonous food onboard Extreme rationing required Cooking equipment on boats is significantly constrained	

**Table 2:** Universal and contextual characteristics of food literacy for migrants in Thailand.

Pillar of Food Literacy	Universal Characteristic	Contextual Characteristic
Plan and Manage	Taking advantage of agriculture seasonality Group/household coordination and time management Efficient food shopping in light of storage and preservation capacity	Prioritizing local raw ingredients Resilience against convenience food Understanding advantages and disadvantages of local market forms Learning to balance work and health Proactive breakfast and lunch preparation to avoid ad-hoc purchase
Select	Discernment of fresh/safe produce Wild food gathering skills Bargaining ability in food markets	Cultivating personal relationships in food retail or food access Recognition of local ingredients
Prepare	Ability to prepare food from scratch Improvisational cooking skills Food preservation Awareness of food safety Efficient use of cooking fuel	Expanded local cooking repertoire Proficiency with local equipment Knowledge of suitable local substitutes for recipe ingredients
Eat	Interest to try new flavors Willingness to eat monotonous food Appreciation of alternative proteins	Enjoyment of local flavors Appreciation of home-made food Creating a social eating environment
